# Enamel Matrix Derivative has No Effect on the Chondrogenic Differentiation of Mesenchymal Stem Cells

**DOI:** 10.3389/fbioe.2014.00029

**Published:** 2014-09-02

**Authors:** Lisanne C. Groeneveldt, Callie Knuth, Janneke Witte-Bouma, Fergal J. O’Brien, Eppo B. Wolvius, Eric Farrell

**Affiliations:** ^1^Department of Oral and Maxillofacial Surgery, Special Dental Care and Orthodontics, Erasmus University Medical Center, Rotterdam, Netherlands; ^2^Tissue Engineering Research Group, Department of Anatomy, Royal College of Surgeons in Ireland, Dublin, Ireland

**Keywords:** enamel matrix derivative, chondrogenesis, mesenchymal stem cells, endochondral ossification, periodontal diseases, differentiation, scaffolds

## Abstract

**Background:** Treatment of large bone defects due to trauma, tumor resection, or congenital abnormalities is challenging. Bone tissue engineering using mesenchymal stem cells (MSCs) represents a promising treatment option. However, the quantity and quality of engineered bone tissue are not sufficient to fill large bone defects. The aim of this study was to determine if the addition of enamel matrix derivative (EMD) improves *in vitro* chondrogenic priming of MSCs to ultimately improve *in vivo* MSC mediated endochondral bone formation.

**Methods:** MSCs were chondrogenically differentiated in 2.0 × 10^5^ cell pellets in medium supplemented with TGFβ3 in the absence or presence of 1, 10, or 100 μg/mL EMD. Samples were analyzed for gene expression of RUNX2, Col II, Col X, and Sox9. Protein and glycoaminoglycan (GAG) production were also investigated via DMB assays, histology, and immunohistochemistry. Osteogenic and adipogenic differentiation capacity were also assessed.

**Results**: The addition of EMD did not negatively affect chondrogenic differentiation of adult human MSCs. EMD did not appear to alter GAG production or expression of chondrogenic genes. Osteogenic and adipogenic differentiation were also unaffected though a trend toward decreased adipogenic gene expression was observed.

**Conclusion**: EMD does not affect chondrogenic differentiation of adult human MSCs. As such the use of EMD in combination with chondrogenically primed MSCs for periodontal bone tissue repair is unlikely to have negative effects on MSC differentiation.

## Introduction

Trauma, tumor resection, or congenital abnormalities can result in large bone defects in the craniomaxillofacial region as well as elsewhere in the body. Treatment options of such defects include the use of autologous or allogenic bone or other substitutes (Arrington et al., 1996; Froum et al., 2001). Autologous bone is preferred clinically; however, harvesting of material can result in secondary site morbidity and an increased risk of infection (Arrington et al., 1996). In addition, tissue availability is limited, increasing the demand for an alternative graft substitute (Meijer et al., 2008).

Tissue engineering represents a promising alternative treatment option for such defects. Mesenchymal stem cells (MSCs), available from tissues including bone marrow and adipose tissue, are multipotent cells that can be differentiated the osteogenic and chondrogenic lineages (Pittenger et al., 1999) making them an attractive cell source for bone tissue engineered constructs.

Multiple approaches have been taken to improve osteogenic differentiation of MSCs, mimicking the process of intramembranous ossification, including manipulating growth factors, scaffolds, and environmental parameters (e.g., oxygen and pressure) (Salgado et al., 2004). Unfortunately, because these bone tissues lack vasculature, necrosis, improper nutrient delivery, and inadequate waste removal occur, ultimately resulting in graft failure.

Bone tissue constructs modeled after the process of endochondral ossification (EO) may result in more promising outcomes as the tissue would be better suited to survive the initial avascular implantation site. During EO, cartilage is formed by chondrogenic differentiation of MSCs *in vitro*. Since chondrocytes reside in an avascular environment, they can survive the initial hypoxic insult following implantation (Coyle et al., 2009). As the chondrocytes mature, become hypertrophic, and apoptose, blood vessels invade and the cartilage rich matrix is mineralized and serves as a template for future bone development. Several groups have produced promising results on the ability of MSCs to guide bone formation along the process of EO *in vivo* (Huang et al., 2006; Farrell et al., 2009, 2011; Gawlitta et al., 2010; Janicki et al., 2010; Scotti et al., 2010; Thompson et al., 2014). van der Stok et al. (2014) demonstrated proof of principle in repairing a long bone defect using this approach. However, despite the promise of this approach, the resulting bone is not sufficient to fill large clinically relevant defects, indicating a need to improve current techniques to optimize bone return. Many researchers have investigated combining MSC with clinically relevant compounds to improve *in vivo* bone formation.

Enamel matrix derivative (EMD) is an extracellular matrix derivative obtained from porcine tooth buds. It contains amelogenin and proteins that belong to the amelogenin family (>90%) (Grandin et al., 2012). It is sold commercially as Emdogain in a single dose syringe dissolved in propylene glycol alginate. Emdogain is used clinically to stimulate the regeneration of periodontal tissues. Combining EMD with surgical periodontal therapy (surgical therapy of the tissue surrounding or encasing teeth) of deep intrabony defects leads to improvement in clinical parameters compared to surgical therapy alone (Froum et al., 2001). Studies have shown that EMD stimulates the proliferation and osteogenic differentiation of MSCs (Narukawa et al., 2007a; Jue et al., 2010; Song et al., 2010; Grandin et al., 2012). However, many groups used only specific proteins that are included in EMD or based their results on cells obtained from animals or cell-lines. The research group of Narukawa found a stimulatory effect of Emdogain on the expression of chondrogenesis-related transcription factors in chondrogenically primed MSCs. Utilizing a chondrogenic cell line, the group also observed an increase in the amount of glycosaminoglycans (GAGs) formed in the extracellular matrix (Narukawa et al., 2007a,b). In an additional study, EMD was shown to increase the proliferation of early chondrocytes derived from rats and inhibited maturation. In more mature chondrocytes, EMD enhanced proliferation and no longer inhibited differentiation (Dean et al., 2002). Due to its clinical relevance and previous evidence indicating an improvement in chondrogenesis, at least on gene expression in a chondrogenic cell line, EMD was hypothesized to improve *in vitro* chondrogenic priming of human MSCs. These chondrogenically primed human MSCs could be implanted in order become hypertrophic and mineralized leading toward bone formation via EO *in vivo*. Assuming this to be the case, it was hypothesized that these primed cells would lead to improved bone formation *in vivo*. The aim of this research was to determine if EMD enhanced chondrogenesis in human MSCs and to determine if EMD improves the quantity and quality of the chondrogenic matrix production. In order to compare with previous research, we also assessed the osteogenic capacity of MSCs in the presence of varying doses of MSCs as well as their adipogenic differentiation capability.

## Materials and Methods

### EMD

Enamel matrix derivative was supplied as a freeze dried preparation by Straumann Company. It was reconstituted in 50 mM acetic acid to 10 mg/mL and further diluted to the working concentrations below in the appropriate culture medium.

### MSC isolation

Mesenchymal stem cells were isolated from three human bone marrow samples aspirated from the greater trochanter major from patients undergoing total hip arthroplasty, after informed consent (METC 2004-142). There were two females (aged 20 and 60) and one male (aged 54). Cells from each donor showed similar growth and differentiation characteristics. Cells were maintained in expansion medium, α-mem (Gibco) containing 10% FCS (Lonza), supplemented with 1 ng/mL FGF2 and 25 μg/mL Ascorbic acid at 37°C and 5% CO_2_ as described previously (Leijs et al., 2012).

### Adipogenesis

Mesenchymal stem cells were cultured in 12-well plates at a density of 7.98 × 10^5^ cells/well. Cells were cultured for 14 days at 37°C and 5% CO_2_ in adipogenic induction medium, DMEM containing 10% FCS, supplemented with 1 μM dexamethasone, 0.2 mM indo-methacin, 0.01 mg/mL insulin, and 0.5 mM 3-isobutyl-l-methyl-xanthine (Sigma). EMD treated samples were cultured in 1, 10, or 100 μg/mL EMD or vehicle alone (0.5 mM acetic acid). Medium was replaced twice a week.

### Osteogenesis

Mesenchymal stem cells were cultured in 12-well plates at a density of 1.14 × 10^4^ cells per well. Cells were cultured for 15–19 days at 37°C and 5% CO_2_ in osteogenic induction medium, high-glucose DMEM (Invitrogen) with addition of 10% FCS, 50 μg/mL gentamycin (Invitrogen), 1.5 μg/mL fungizone (Invitrogen), 10 mM glycerol 2-phosphate (Sigma), 0.1 μM dexamethasone (Sigma), and 0.1 mM ascorbic acid (Sigma). EMD treated samples were cultured in 1, 10, or 100 μg/mL EMD or vehicle. Medium was replaced twice a week. Samples were harvested at the latest point prior to detachment of the cells from the surface of the tissue culture plastic, as occurs during osteogenic differentiation in monolayer. This varied from 15–19 days between donors.

### Chondrogenesis

Mesenchymal stem cells were cultured for 21 or 35 days in pellets of 2.0 × 10^5^ cells in chondrogenic medium, high-glucose DMEM supplemented with 50 μg/mL gentamycin (Invitrogen), 1.5 μg/mL fungizone (Invitrogen), 1 mM sodium pyruvate (Invitrogen), 40 μg/mL proline (Sigma), 1:100v/v insulin-transferrin-selenium (ITS; BD Biosciences), 10 ng/mL transforming growth factor β1 (R&D Systems), 25 μg/mL ascorbic acid (Sigma), and 100 nM dexamethasone (Sigma). EMD treated samples were cultured in 1, 10, or 100 μg/mL EMD or vehicle alone. Medium was replaced twice a week.

### Oil red O staining

Lipid droplets were stained by Oil Red O. Cells in monolayer were washed in 0.9% NaCl and fixed for 1 h in 4% paraformaldehyde. Cells were stained with Oil Red O (0.3% w/v in distilled water; Sigma) for 10–15 min and washed with distilled water. Cells were mounted with Vectamount.

### von Kossa staining

Cells in monolayer were washed in 0.9% NaCl, fixed with 4% formaldehyde for 1 h and stained with von Kossa staining. Cells were incubated in 5% silver nitrate and placed on a light box for 15 min. Excess silver nitrate was washed using distilled water and cells were placed on a light box for another 10 min. Cells were washed in distilled water and counterstained with thionine for 5 min. Cells were dehydrated in 70% (10 s), 96% (30 s), and 100% ethanol (2 min) and mounted with Vectamount.

### Scaffold seeding

Collagen-glycosaminoglycan (Collagen-GAG) scaffolds were cut in 8 mm squares, placed in 6-well plates coated with 2% agarose (LE- analytical grade, Promega). Scaffolds were seeded with 5 × 10^5^ cells in 150 μL culture medium on one side, incubated for 30 min then overturned and seeded again with the same cell number and volume. After another 30 min, the well was filled with 3 mL of culture medium. Constructs were cultured in chondrogenic medium with the addition or absence of 10 ng/mL transforming growth factor-β 1 (TGF-β1) and/or 100 μg/mL EMD. Samples were cultured at 37°C and 5% CO_2._

### Gene expression analysis

RNA was isolated from chondrogenic pellets by homogenizing samples with a Eppendorf-potter in 350 μL RNAbee (Freund Can Company). Adipogenic and osteogenic primed MSCs cultured in monolayers, 2-wells were combined in 300 μL RNAbee. RNA isolation, cDNA synthesis, and measurement of gene expression levels on 8–15 ng cDNA were performed as described before (Verseijden et al., 2009; Leijs et al., 2012). Primers and probes used for alkaline phosphatase (ALPL), Gamma-carboxyglutamic acid-containing protein (BGLAP), Integrin-binding sialoprotein (IBSP), Collagen type I (COLI), Peroxisome proliferator-activated receptor γ (PPARγ), Fatty acid-binding protein 4 (FABP4), Runt-related transcription factor 2 (RUNX2), Collagen type II (COL II), Collagen type X (COL X), Sex determining region Y-box 9 (SOX 9), and Glyceraldehyde-3-phosphate dehydrogenase (GAPDH) are represented in Table S1 in Supplementary Material.

### Glycosaminoglycan quantification

Pellets and scaffolds were digested in 150 μL papaine digestion solution in combination with 150 μL sodium citrate buffer. GAGs were measured and adjusted to the amount of DNA present in each pellet or scaffold as described before using heparin (Leo Pharmaceutical Products BV), RNAse (Ribonuclease type III-A; Sigma), and ethidium bromide (GibcoBR1) (Clockaerts et al., 2011).

### Histological preparation

Pellets and scaffolds were fixed in 4% paraformaldehyde for 1 h, embedded in liquid paraffin wax, and cut into 5 μm sections using a microtome (Leica RM2135). Sections were placed onto SuperStar^®^ microscope slides and de-waxed by soaking sequentially in xylene and 100, 96, and 70% ethanol (5 min each).

### Glycosaminoglycan and H&E staining

Glycoaminoglycan formation was determined by 0.1% safranin O staining and cell morphology was determined utilizing H&E staining. Stainings were performed as described previously (Narcisi et al., 2012; de Vries-van Melle et al., 2013).

### Immunohistochemical staining for collagen type II

Antigen retrieval was performed using 0.1% pronase and 1% hyaluronidase. Sections were incubated with 1:100 mouse monoclonal antibody against collagen type II and stained by an ALPL substrate as described before (Narcisi et al., 2012).

### Statistics

Data are presented as mean values ±SD. Statistical analysis was carried out using repeated measures ANOVA test followed by Tukey *post hoc* correction using a statistical software package (Prism 5.00, Graphpad Software). Results were considered statistically significant at *p* < 0.05.

## Results

### EMD does not affect the osteogenic differentiation capacity of human MSCs

**Figure 1:**
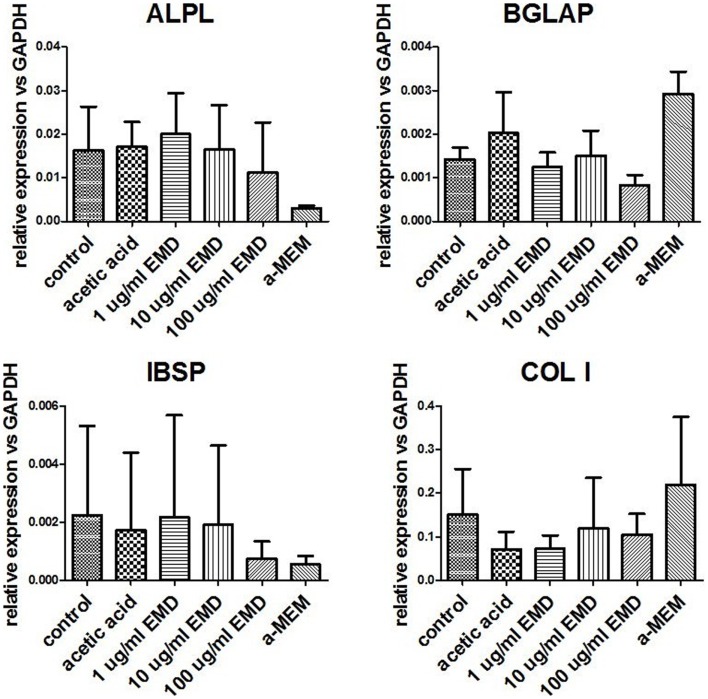
**Measurement of gene expression levels for osteogenic genes**. Gene expression was measured in MSCs cultured in osteogenic medium for 15–19 days. Data represent fold changes of target genes relative to the housekeeping gene GAPDH. Values represent the mean ± SD for samples from three donors.

**Figure 2:**
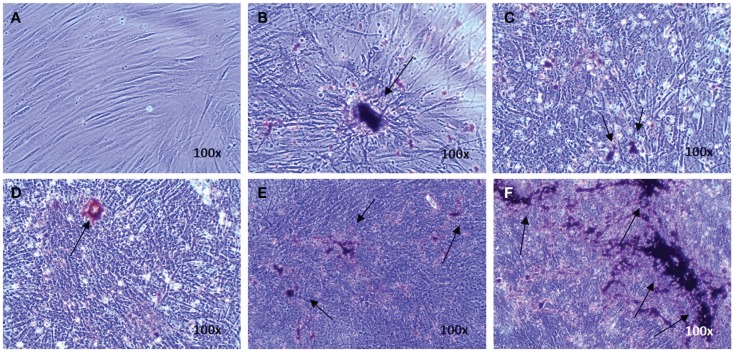
**Evidence of osteogenic differentiation of MSCs exposed to osteogenic factors for 19 days**. Images represent von Kossa staining for MSCs of one donor cultured in the non-differentiation medium α-MEM as a negative control **(A)** MSCs cultured in osteogenic differentiation only **(B)**, MSCs cultured in osteogenic differentiation medium in presence of the vehicle **(C)**, and MSCs cultured in osteogenic differentiation medium in presence of 1, 10, or 100 μg/mL EMD **(D–F)** (100× magnification). Arrows indicate calcium phosphate-containing nodules.

Osteogenic genes ALPL, BGLAP, IBSP, and COL I were analyzed after 15–19 days by real-time PCR. No differences were observed between osteogenic control, vehicle, and the different doses of EMD (*p*-values respectively 0.1600, 0.2578, 0.6016, and 0.5673; Figure 1). Despite inter-donor variability, no differences were observed in the amount of calcium phosphate-nodules formed at the macroscopic level (Figure 2). This suggests that EMD had no effect on the osteogenic differentiation of MSCs.

### EMD has no effect on the adipogenic differentiation of human MSCs

**Figure 3:**
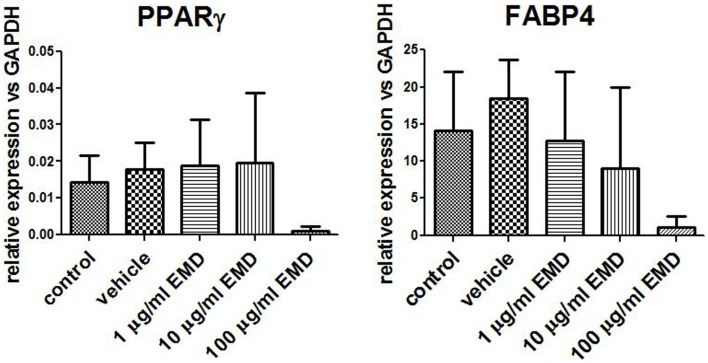
**Measurement of gene expression levels for PPARγ and FABP4**. Gene expression was measured in MSCs cultured in adipogenic differentiation medium for 14 days with addition of vehicle or different doses of EMD (1, 10, or 100 μg/mL). Data represent fold changes of target genes relative to the housekeeping gene GAPDH. Values represent the mean ± SD for samples from three donors.

**Figure 4:**
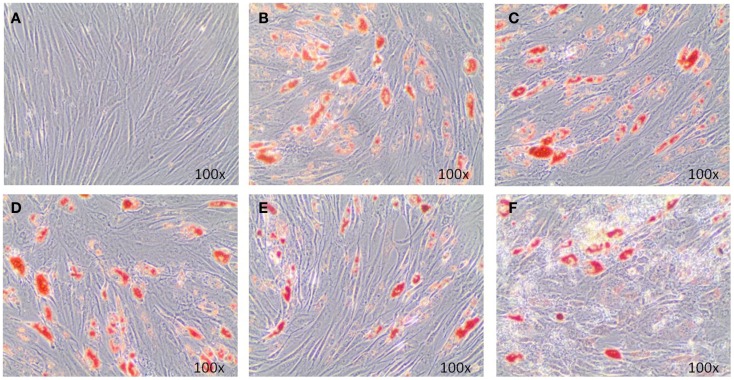
**Oil Red O staining illustrates adipogenic differentiation of MSCs exposed to adipogenic factors for 14 days**. Images represent MSCs cultured in the non-differentiation medium α-MEM **(A)**, MSCs cultures in adipogenic differentiation medium only **(B)**, MSCs cultured in adipogenic differentiation medium in addition of vehicle only (0.5 mM acetic acid) **(C)**, and MSCs cultured in adipogenic differentiation medium in addition of 1, 10, or 100 μg/mL EMD **(D–F)** (100× magnification).

Adipogenic genes, FABP4 and PPARγ, were investigated for three donors by real-time PCR after 14 days to determine the role of EMD on adipogenesis. Cells cultured in the high dose EMD (100 μg/mL) showed a trend toward inhibition of gene expression compared to vehicle and adipogenic control. However, given the large inter-donor variability, this difference was not statistically significant for FABP4 (*p* = 0.4835) or PPARγ (*p* = 0.1063; Figure 3). The effects of vehicle and EMD on adipogenic differentiation were also assessed by Oil Red O staining of fat-containing droplets. No cells cultured in the expansion medium (used as a negative control) showed evidence of fat-containing droplets (Figure 4A). When MSCs were cultured in all other treatment conditions, cells positively stained in all conditions (Figures 4B–F). Staining was slightly reduced in the high dose EMD (100 μg/mL) compared to adipogenic control or vehicle across all donors and wells. This suggests, together with the results for FABP4 and PPARγ, a potentially inhibitory effect of EMD on adipogenic differentiation of MSCs at the highest dose. However, this effect was minimal as determined by staining.

### EMD does not affect the chondrogenic differentiation capacity of human MSCs

**Figure 5:**
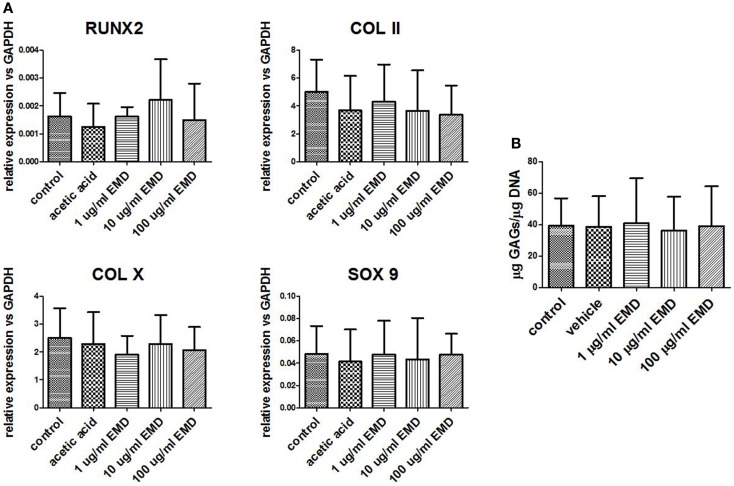
**Gene expression levels of chondrogenic genes following differentiation**. **(A)** Gene expression was measured in MSCs cultured in chondrogenic differentiation medium treated with vehicle only or different doses of EMD (1, 10, or 100 μg/mL) for 35 days. Data represent fold changes of target genes relative to the housekeeping gene GAPDH. Values represent the mean ± SD for samples from three donors. **(B)** Quantification of GAGs in MSCs cultured with chondrogenic factors. Data represent amount of GAGs normalized to DNA content in each pellet.

Chondrogenically primed cell pellets were analyzed by real-time PCR after 21 days for three donors. Four different chondrogenic genes were analyzed; COL II, COL X, SOX 9, and RUNX2 (Figure 5). After treatment with vehicle only or EMD, no statistical significant differences in COL II (*p* = 0.0538), COL X (*p* = 0.2457), SOX 9 (*p* = 0.7458), or RUNX2 (*p* = 0.5863) mRNA levels were observed between groups.

**Figure 6:**
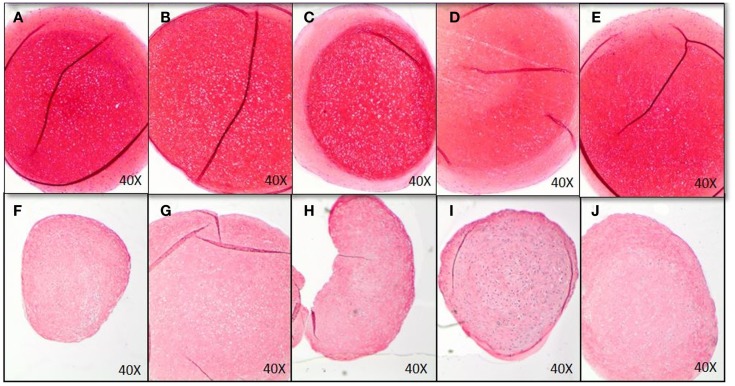
**Histological analysis of chondrogenic differentiation of MSCs exposed to chondrogenic factors for 35 days**. Images represent MSCs cultured in chondrogenic differentiation medium only **(A,F)**, MSCs cultured in chondrogenic differentiation medium in addition of vehicle only **(B,G)**, and MSCs cultured in chondrogenic differentiation medium in addition of 1 μg/mL **(C,H)**, 10 μg/mL **(D,I)**, or 100 μg/mL EMD **(E,J)**. GAGs were stained by Safranin O **(A–E)**, COL II immunohistochemistry was performed for images **(F–J)**.

GAG-production measured in control was approximately 40 μg GAG per microgram DNA. There was no effect of EMD at any concentration on the quantity of GAG production (*p* = 0.8989; Figure 5B). Following 35 days of culture in chondrogenic medium, or in the presence of vehicle, or EMD, chondrogenic pellets were stained with safranin O (Figures 6A–E). Immunohistochemical staining for COL II was also performed on these pellets (Figures 6F–J). All pellets demonstrated high quantities of GAGs stained by safranin O and collagen type II. However, no differences in staining were observed between pellets in the chondrogenic control conditions or in the presence of different doses of EMD (1, 10, or 100μg/mL).

### MSCs in 3D culture

**Figure 7:**
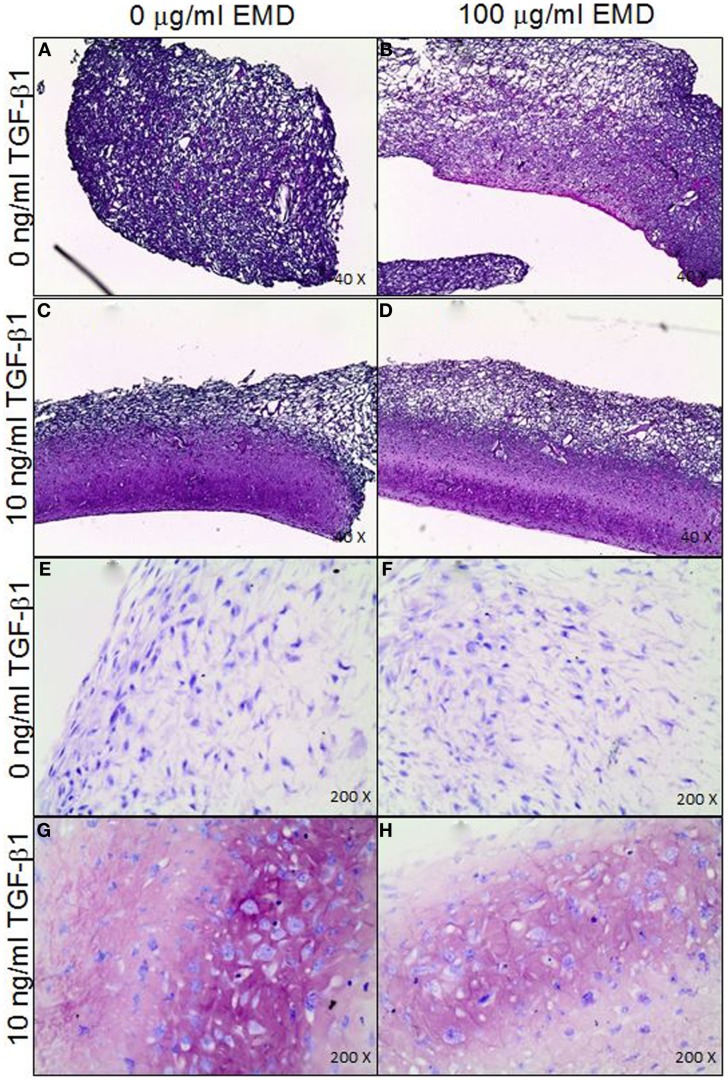
**(A–D)** Distribution of MSCs through the collagen-GAG scaffolds cultured under the four different conditions (H&E staining). **(E–H)** Staining for GAGs produced by MSCs seeded on collagen-GAG scaffolds cultured whether or not in presence of TGF-β and/or EMD (thionine staining).

**Figure 8:**
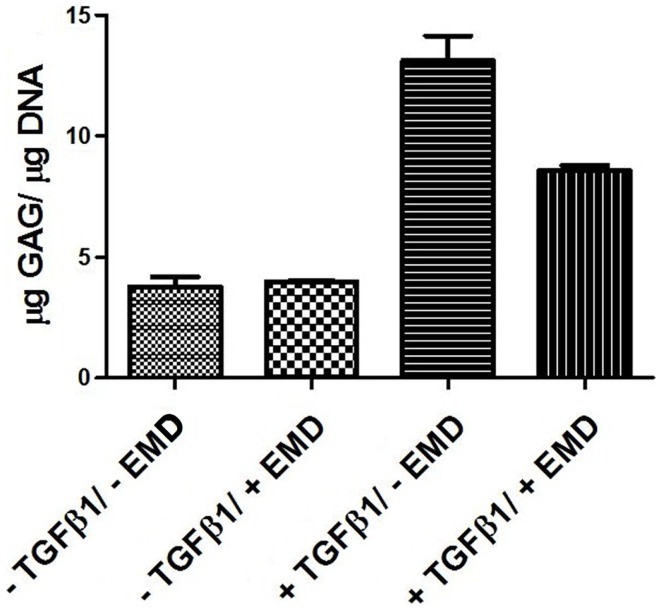
**Quantification of GAGs in MSCs seeded on scaffolds cultured with chondrogenic factors for 21 days (two samples from one donor for each condition)**.

In order to assess the effects of EMD on the cell distribution and chondrogenic differentiation in a 3D environment, two collagen-GAG scaffolds were seeded with human MSCs and cultured in the presence or absence of TGFβ1 (10 ng/mL) and/or EMD (100 μg/mL). Hematoxylin and eosin staining demonstrated similar cellular distribution in both conditions (Figures 7A–D). Thionine staining illustrated the presence of GAGs in both conditions (Figures 7E–H). Upon quantification of the amount of GAG production in two scaffolds per condition, less GAG/DNA was produced in the TGFβ1 + EMD condition (Figure 8). As this was only performed with cells from one donor, it was not possible to statistically analyze these results.

## Discussion

Treatment of large bone defects is challenging. Current treatment options such as autologous bone or bone substitutes are often accompanied by limitations and serious complications, highlighting the necessity for an alternative treatment option to be developed (Arrington et al., 1996; Meijer et al., 2008; Vahabi et al., 2012; Wiggins et al., 2012). Reports on the ability of EMD to improve MSC osteogenesis are mixed (Narukawa et al., 2007b; Gawlitta et al., 2010; Jue et al., 2010; Qu et al., 2010; Grandin et al., 2012) while little is known about the effects of EMD on chondrogenesis of human MSCs. Bone formation utilizing EO, in which MSCs are chondrogenically differentiated *in vitro*, and implanted, forming bone *in vivo*, represents a promising new avenue of research in the field of bone tissue engineering (Gawlitta et al., 2010). We hypothesized that, given the reported abilities of EMD to improve cell proliferation, migration, and differentiation (particularly osteogenically) (Narukawa et al., 2007a; Jue et al., 2010; Qu et al., 2010; Song et al., 2010), EMD might also improve the chondrogenic priming of human MSCs. In this study, we focused on chondrogenic differentiation of adult human MSCs as a first step to tissue engineering bone via the process of EO. In order to put the work in the context of prior research, we also assessed osteogenic and adipogenic differentiation of these cells in the presence of EMD.

No differences were observed in GAG production nor in COL II expression in any of the conditions. While the group of Narukawa found an upregulation of COL II, COL X, and SOX 9, as well as increased GAG production following chondrogenic treatment of the ATDC5 hypertrophic cell line in the presence of Emdogain (Narukawa et al., 2007a), we observed no effects on chrondrogenic differentiation in primary human MSCs. Given the natural tendency of these teratoma derived ATDC5 cells to progress along the chondrogenic lineage toward hypertrophy, it is hard to directly compare the two cell types. The effect of EMD on cell migration and chondrogenesis in a 3D environment, a collagen-GAG scaffold, was also analyzed in this study. This was only performed using cells from a single donor on two scaffolds per condition. On histology, no differences were observed between chondrogenically treated groups. However, while chondrogenisis did occur, there was a trend toward decreased GAG production in the EMD treated samples. This experiment would require repetition with MSCs from several donors to confirm if this is the case.

We observed no effect of EMD at any dose on the ability of cells extracellular matrix production or on the gene level when stimulated osteogenically. This is in agreement with the work of some other groups (van den Dolder et al., 2006; Mrozik et al., 2012). However, other groups also stated EMD, or components of it, stimulated the differentiation of MSCs toward osteocytes (Keila et al., 2004; Itoh et al., 2006; Narukawa et al., 2007b; Amin et al., 2012; Ramis et al., 2012). These groups utilized both cell-lines and rat derived MSCs, as well as only selective proteins found in EMD, which may explain the differences observed. Considering published work and our results, we have no evidence to support the idea that EMD would negatively influence osteogenic differentiation of human MSCs. The *in vivo* effects of EMD on ossification remain unclear. Some groups reported enhanced bone induction *in vivo* in both animals and humans (Hammarstrom et al., 1997; Boyan et al., 2000; Jaiswal and Deo, 2013) while others showed no effect of EMD on the formation of mineralized bone (Schneider et al., 2011). Yagi showed that EMD inhibits RANKL expression, resulting in inhibited osteoclast formation, the cells that are responsible for bone resorption (Yagi et al., 2009). The variability in these results could be caused by factors such as biological characteristics of the defect and patient variability (Venezia et al., 2004). However, these results are based on bone formation by surrounding cells instead of implanted chondrogenically primed cells. It is difficult to extrapolate the results observed in this study to the *in vivo*/clinical situation. We observed a mild trend toward inhibition of adipogenic differentiation at the highest dose of EMD on human MSCs. No tests have been performed to determine the effects of EMD on adipogenic differentiation of MSCs previously. The decreasing trend toward adipogenic differentiation of MSCs, in this proposed application, could be considered a positive outcome suggesting undesirable fat tissue formation is unlikely.

Enamel matrix derivative does not appear to effect the multilineage differentiation of human MSCs. There may be a slight inhibitory effect of EMD, at the highest dose, on adipogenesis. However, this was not proven to be statistically significant. While this work suggests that EMD would not increase the chondrogenic potential of MSCs, which could be utilized in a bone tissue construct via EO for the treatment of large bone defects, there is also no evidence that bone formation would be inhibited if EMD was used in combination with MSCs for the repair of minor bone defects for periodontal tissue repair.

## Conflict of Interest Statement

The authors declare that the research was conducted in the absence of any commercial or financial relationships that could be construed as a potential conflict of interest.

## Supplementary Material

The Supplementary Material for this article can be found online at http://www.frontiersin.org/Journal/10.3389/fbioe.2014.00029/abstract

## References

[B1] Amin H. D. Olsen I. Knowles J. C. Donos N. (2012). Differential effect of amelogenin peptides on osteogenic differentiation in vitro: identification of possible new drugs for bone repair and regeneration. Tissue Eng. Part A 18, 1193–1202 10.1089/ten.TEA.2011.0375 22320389

[B2] Arrington E. D. Smith W. J. Chambers H. G. Bucknell A. L. Davino N. A. (1996). Complications of iliac crest bone graft harvesting. Clin. Orthop. Relat. Res. 329, 300–309 10.1097/00003086-199608000-00037 8769465

[B3] Boyan B. D. Weesner T. C. Lohmann C. H. Andreacchio D. Carnes D. L. Dean D. D. (2000). Porcine fetal enamel matrix derivative enhances bone formation induced by demineralized freeze dried bone allograft in vivo. J. Periodontol. 71, 1278–1286 10.1902/jop.2000.71.8.1278 10972643

[B4] Clockaerts S. Bastiaansen-Jenniskens Y. M. Feijt C. Verhaar J. A. Somville J. De Clerck L. S. (2011). Peroxisome proliferator activated receptor alpha activation decreases inflammatory and destructive responses in osteoarthritic cartilage. Osteoarthr. Cartil. 19, 895–902 10.1016/j.joca.2011.03.010 21458577

[B5] Coyle C. H. Izzo N. J. Chu C. R. (2009). Sustained hypoxia enhances chondrocyte matrix synthesis. J. Orthop. Res. 27, 793–799 10.1002/jor.20816 19051248 PMC6548435

[B6] de Vries-van Melle M. Narcisi R. Kops N. Koevoet W. Bos P. K. Murphy J. M. (2013). Chondrogenesis of mesenchymal stem cells in an osteochondral environment is mediated by the subchondral bone. Tissue Eng. Part A 20, 23–33 10.1089/ten.TEA.2013.0080 23980750 PMC3875203

[B7] Dean D. D. Lohmann C. H. Sylvia V. L. Cochran D. L. Liu Y. Boyan B. D. (2002). Effect of porcine fetal enamel matrix derivative on chondrocyte proliferation, differentiation, and local factor production is dependent on cell maturation state. Cells Tissues Organs 171, 117–127 10.1159/000063705 12097834

[B8] Farrell E. Both S. K. Odorfer K. I. Koevoet W. Kops N. O’Brien F. J. (2011). In-vivo generation of bone via endochondral ossification by in-vitro chondrogenic priming of adult human and rat mesenchymal stem cells. BMC Musculoskelet. Disord. 12:31 10.1186/1471-2474-12-31 21281488 PMC3045394

[B9] Farrell E. van der Jagt O. P. Koevoet W. Kops N. van Manen C. J. Hellingman C. A. (2009). Chondrogenic priming of human bone marrow stromal cells: a better route to bone repair? Tissue Eng. Part C Methods 15, 285–295 10.1089/ten.tec.2008.0297 19505182

[B10] Froum S. J. Weinberg M. A. Rosenberg E. Tarnow D. (2001). A comparative study utilizing open flap debridement with and without enamel matrix derivative in the treatment of periodontal intrabony defects: a 12-month re-entry study. J. Periodontol. 72, 25–34 10.1902/jop.2001.72.1.25 11210070

[B11] Gawlitta D. Farrell E. Malda J. Creemers L. B. Alblas J. Dhert W. J. (2010). Modulating endochondral ossification of multipotent stromal cells for bone regeneration. Tissue Eng. Part B Rev. 16, 385–395 10.1089/ten.TEB.2009.0712 20131956

[B12] Grandin H. M. Gemperli A. C. Dard M. (2012). Enamel matrix derivative: a review of cellular effects in vitro and a model of molecular arrangement and functioning. Tissue Eng. Part B Rev. 18, 181–202 10.1089/ten.TEB.2011.0365 22070552

[B13] Hammarstrom L. Heijl L. Gestrelius S. (1997). Periodontal regeneration in a buccal dehiscence model in monkeys after application of enamel matrix proteins. J. Clin. Periodontol. 24, 669–677 10.1111/j.1600-051X.1997.tb00248.x 9310871

[B14] Huang J. I. Durbhakula M. M. Angele P. Johnstone B. Yoo J. U. (2006). Lunate arthroplasty with autologous mesenchymal stem cells in a rabbit model. J. Bone Joint Surg. 88A, 744–752 10.2106/JBJS.E.00669 16595464

[B15] Itoh N. Kasai H. Ariyoshi W. Harada E. Yokota M. Nishihara T. (2006). Mechanisms involved in the enhancement of osteoclast formation by enamel matrix derivative. J. Periodont. Res. 41, 273–279 10.1111/j.1600-0765.2005.00868.x 16827720

[B16] Jaiswal R. Deo V. (2013). Evaluation of the effectiveness of enamel matrix derivative, bone grafts, and membrane in the treatment of mandibular class II furcation defects. Int. J. Periodontics Restorative Dent. 33, e58–e64 10.11607/prd.1428 23484181

[B17] Janicki P. Kasten P. Kleinschmidt K. Luginbuehl R. Richter W. (2010). Chondrogenic pre-induction of human mesenchymal stem cells on beta-TCP: enhanced bone quality by endochondral heterotopic bone formation. Acta Biomater. 6, 3292–3301 10.1016/j.actbio.2010.01.037 20123138

[B18] Jue S. S. Lee W. Y. Kwon Y. D. Kim Y. R. Pae A. Lee B. (2010). The effects of enamel matrix derivative on the proliferation and differentiation of human mesenchymal stem cells. Clin. Oral Implants Res. 21, 741–746 10.1111/j.1600-0501.2009.01901.x 20636728

[B19] Keila S. Nemcovsky C. E. Moses O. Artzi Z. Weinreb M. (2004). In vitro effects of enamel matrix proteins on rat bone marrow cells and gingival fibroblasts. J. Dent. Res. 83, 134–138 10.1177/154405910408300210 14742651

[B20] Leijs M. J. van Buul G. M. Lubberts E. Bos P. K. Verhaar J. A. Hoogduijn M. J. (2012). Effect of arthritic synovial fluids on the expression of immunomodulatory factors by mesenchymal stem cells: an explorative in vitro study. Front. Immunol. 3:231 10.3389/fimmu.2012.00231 22876244 PMC3410447

[B21] Meijer G. J. de Bruijn J. D. Koole R. van Blitterswijk C. A. (2008). Cell based bone tissue engineering in jaw defects. Biomaterials 29, 3053–3061 10.1016/j.biomaterials.2008.03.012 18433864

[B22] Mrozik K. M. Gronthos S. Menicanin D. Marino V. Bartold P. M. (2012). Effect of coating Straumann bone ceramic with emdogain on mesenchymal stromal cell hard tissue formation. Clin. Oral Investig. 16, 867–878 10.1007/s00784-011-0558-3 21584694

[B23] Narcisi R. Signorile L. Verhaar J. A. Giannoni P. van Osch G. J. (2012). TGFbeta inhibition during expansion phase increases the chondrogenic re-differentiation capacity of human articular chondrocytes. Osteoarthr. Cartil. 20, 1152–1160 10.1016/j.joca.2012.06.010 22772045

[B24] Narukawa M. Suzuki N. Takayama T. Shoji T. Otsuka K. Ito K. (2007a). Enamel matrix derivative stimulates chondrogenic differentiation of ATDC5 cells. J. Periodont. Res. 42, 131–137 10.1111/j.1600-0765.2006.00926.x 17305871

[B25] Narukawa M. Suzuki N. Takayama T. Yamashita Y. Otsuka K. Ito K. (2007b). Enamel matrix derivative stimulates osteogenesis- and chondrogenesis-related transcription factors in C3H10T1/2 cells. Chin. J. Biochem. Biophys. 39, 1–7 10.1111/j.1745-7270.2007.00250.x 17213952

[B26] Pittenger M. F. Mackay A. M. Beck S. C. Jaiswal R. K. Douglas R. Mosca J. D. (1999). Multilineage potential of adult human mesenchymal stem cells. Science 284, 143–147 10.1126/science.284.5411.143 10102814

[B27] Qu Z. Laky M. Ulm C. Matejka M. Dard M. Andrukhov O. (2010). Effect of Emdogain on proliferation and migration of different periodontal tissue-associated cells. Oral Surg. Oral Med. Oral Pathol. Oral Radiol. Endod. 109, 924–931 10.1016/j.tripleo.2010.01.007 20399692

[B28] Ramis J. M. Rubert M. Vondrasek J. Gaya A. Lyngstadaas S. P. Monjo M. (2012). Effect of enamel matrix derivative and of proline-rich synthetic peptides on the differentiation of human mesenchymal stem cells toward the osteogenic lineage. Tissue Eng. Part A 18, 1253–1263 10.1089/ten.tea.2011.0404 22429009

[B29] Salgado A. J. Coutinho O. P. Reis R. L. (2004). Bone tissue engineering: state of the art and future trends. Macromol. Biosci. 4, 743–765 10.1002/mabi.200400026 15468269

[B30] Schneider D. Weber F. E. Hammerle C. H. Feloutzis A. Jung R. E. (2011). Bone regeneration using a synthetic matrix containing enamel matrix derivate. Clin. Oral Implants Res. 22, 214–222 10.1111/j.1600-0501.2010.01985.x 21223377

[B31] Scotti C. Tonnarelli B. Papadimitropoulos A. Scherberich A. Schaeren S. Schauerte A. (2010). Recapitulation of endochondral bone formation using human adult mesenchymal stem cells as a paradigm for developmental engineering. Proc. Natl. Acad. Sci. U.S.A. 107, 7251–7256 10.1073/pnas.1000302107 20406908 PMC2867676

[B32] Song Z. C. Shu R. Zhang X. L. (2010). Cellular responses and expression profiling of human bone marrow stromal cells stimulated with enamel matrix proteins in vitro. Cell Prolif. 43, 84–94 10.1111/j.1365-2184.2009.00656.x 19922487 PMC6496572

[B33] Thompson E. M. Matsiko A. Farrell E. Kelly D. J. O’Brien F. J. (2014). Recapitulating endochondral ossification: a promising route to in vivo bone regeneration. J. Tissue Eng. Regen. Med. 10.1002/term.1918 24916192

[B34] Vahabi S. Amirizadeh N. Shokrgozar M. A. Mofeed R. Mashhadi A. Aghaloo M. (2012). A comparison between the efficacy of Bio-Oss, hydroxyapatite tricalcium phosphate and combination of mesenchymal stem cells in inducing bone regeneration. Chang Gung Med. J. 35, 28–37 22483425 10.4103/2319-4170.106169

[B35] van den Dolder J. Vloon A. P. Jansen J. A. (2006). The effect of Emdogain on the growth and differentiation of rat bone marrow cells. J. Periodont. Res. 41, 471–476 10.1111/j.1600-0765.2006.00894.x 16953824

[B36] van der Stok J. Koolen M. K. Jahr H. Kops N. Waarsing J. H. Weinans H. (2014). Chondrogenically differentiated mesenchymal stromal cell pellets stimulate endochondral bone regeneration in critical-sized bone defects. Eur. Cell. Mater. 27, 137–148 24554271 10.22203/ecm.v027a11

[B37] Venezia E. Goldstein M. Boyan B. D. Schwartz Z. (2004). The use of enamel matrix derivative in the treatment of periodontal defects: a literature review and meta-analysis. Crit. Rev. Oral Biol. Med. 15, 382–402 10.1177/154411130401500605 15574680

[B38] Verseijden F. Jahr H. Posthumus-van Sluijs S. J. Ten Hagen T. L. Hovius S. E. Seynhaeve A. L. (2009). Angiogenic capacity of human adipose-derived stromal cells during adipogenic differentiation: an in vitro study. Tissue Eng. Part A 15, 445–452 10.1089/ten.tea.2007.0429 18652540

[B39] Wiggins A. Austerberry R. Morrison D. Ho K. M. Honeybul S. (2012). Cranioplasty with custom-made titanium plates – fourteen years experience. Neurosurgery 72, 248–256 10.1227/NEU.0b013e31827b98f3 23149967

[B40] Yagi Y. Suda N. Yamakoshi Y. Baba O. Moriyama K. (2009). In vivo application of amelogenin suppresses root resorption. J. Dent. Res. 88, 176–181 10.1177/0022034508329451 19278991

