# Supplementary material for: Enamel Matrix Derivative has No Effect on the Chondrogenic Differentiation of Mesenchymal Stem Cells
Source: Front Bioeng Biotechnol. 2014 Sep 2;2:29. doi: 10.3389/fbioe.2014.00029 (PMC4151337; doi:10.3389/fbioe.2014.00029)
Supplement: Supplementary file 1 [file Table1.DOCX]

|  | ALPL  BGLAP  IBSP  COL I  PPARγ  FABP4  RUNX2  COL II      COL X      SOX 9      GAPDH | Fw  FAM  Rv  Fw  FAM  Rv  Fw  FAM  Rv  Fw  FAM  Rv  Fw  FAM  Rv  Fw  FAM  Rv  Fw  FAM  Rv  Fw  FAM  Rv  Fw  FAM  Rv  Fw  FAM  Rv  Fw  FAM  Rv | GACCCTTGACCCCCACAAT  TGGACTACCTATTGGGTCTCTTCGAGCCA  GCTCGTACTGCATGTCCCCT  GAAGCCCAGCGGTGC  TGGACACAAAGGCTGCACCTTTGCT  CACTACCTCGCTGCCCTCC  TGCCTTGAGCCTGCTTCC  CTCCAGGACTGCCAGAGGAAGCAATCA  GCAAAATTAAAGCAGTCTTCATTTTG  CAGCCGCTTCACCTACAGC  CCGGTGTGACTCGTGCAGCCATC  TTTTGTATTCAATCACTGTCTTGCC  AGGGCGATCTTGACAGGAAA    TCTCCCATCATTAAGGAATTCATG  TCAGTGTGAATGGGGATGTGAT    TTCAATGCGAACTTCAGTCCAG  GCCTTCAAGGTGGTAGCC  CCACAGTCCCATCTGGTACCTCTCCG  CGTTACCCGCCATGACAGTA  GGCAATAGCAGGTTCACGTACA  CCGGTATGTTTCGTGCAGCCATCCT  CGATAACAGTCTTGCCCCACTT  CAAGGCACCATCTCCAGGAA  TCCAGCACGCAGAATCCATCTGA  AAAGGGTATTTGTGGCAGCATATT  CAACGCCGAGCTCAGCA  TGGGCAAGCTCTGGAGACTTCTGAACG  TCCACGAAGGGCCGC  ATGGGGAAGGTGAAGGTCG  CGCCCAATACGACCAAATCCGTTGAC  TAAAAGCAGCCCTGGTGACC |
| --- | --- | --- | --- |

*Table 1: Sequences of primers and FAM-labelled Taqman probes used in order to define expression of the gene. FW, forward; RV, reverse; FAM, FAM-labelled Taqman Probe.*
